# Toll-Like Receptor Ligands Induce Expression of the Costimulatory Molecule CD155 on Antigen-Presenting Cells

**DOI:** 10.1371/journal.pone.0054406

**Published:** 2013-01-17

**Authors:** Neha Kamran, Yoshimi Takai, Jun Miyoshi, Subhra K. Biswas, Justin S. B. Wong, Stephan Gasser

**Affiliations:** 1 Immunology Programme, Department of Microbiology, National University of Singapore, Singapore, Singapore; 2 National University of Singapore Graduate School for Integrative Sciences and Engineering, National University of Singapore, Singapore, Singapore; 3 Division of Molecular and Cellular Biology, Department of Biochemistry and Molecular Biology, Kobe University Graduate School of Medicine, Kobe, Japan; 4 Department of Molecular Biology, Osaka Medical Center for Cancer and Cardiovascular Diseases, Osaka, Japan; 5 Singapore Immunology Network, Singapore; National Jewish Health and University of Colorado School of Medicine, United States of America

## Abstract

Genotoxic stress and RAS induce the expression of CD155, a ligand for the immune receptors DNAM-1, CD96 and TIGIT. Here we show that antigen-presenting cells upregulate CD155 expression in response to Toll-like receptor activation. Induction of CD155 by Toll-like receptors depended on MYD88, TRIF and NF-κB. In addition, IRF3, but not IRF7, modulated CD155 upregulation in response to TLR3 signals. Immunization of CD155-deficient mice with OVA and the TLR9 agonist CpG resulted in increased OVA-specific IgG2a/c titers when compared to wild type mice. Splenocytes of immunized CD155-deficient mice secreted lower levels of IL-4 and fewer IL-4 and GATA-3 expressing CD4^+^ T cells were present in the spleen of *Cd155^−/−^* mice. Our data suggest that CD155 regulates T_h_2 differentiation. Targeting of CD155 in immunization protocols using peptides may represent a promising new approach to boost protective humoral immunity in viral vaccines.

## Introduction

Toll-like receptors (TLRs) are key sensors of the innate immune system that recognize conserved microbial domains known as pathogen-associated molecular patterns (PAMPs) such as LPS, flagellin and double-stranded RNA [1], [2]. Upon activation, TLRs recruit the proximal adapter molecule myeloid differentiation factor 88 (MYD88) and/or the Toll/IL-1 receptor domain-containing adaptor inducing IFN-β (TRIF) and activate various downstream pathways including mitogen-activated protein kinases (MAPKs), nuclear factor-κB (NF-κB) and interferon regulatory factors (IRFs). Most TLRs depend on MYD88 for their functions, whereas TLR3 requires TRIF for its activity and TLR4 activates both MYD88-dependent and TRIF-dependent responses. TLRs promote the function of antigen-presenting cells (APCs) by enhancing their antigen-presenting activity, cytokine production and expression of costimulatory molecules [3].

Activation of naïve CD4^+^ T cells by APCs leads to their differentiation into different subsets depending on the co-stimulatory signals and cytokines expressed by the APCs. T helper (T_h_) 1 cells preferentially produce IFN-γ and stimulate B cells to produce IgG2a/c antibodies, while T_h_2 cells secrete IL-4, IL-5 and IL-13 that are critical for IgE production [4]. It was reported that TLR4 and TLR9 agonists induce the secretion of proinflammatory cytokines such as IL-12 and IL-18, which support T_h_1 cell differentiation, while TLR2-induced expression of ICOSL was shown to promote T_h_2 differentiation [5], [6], [7]. However, the TLR-induced costimulatory signals involved in the regulation of T_h_1 and T_h_2 differentiation are not well characterized.

CD155, also known as Necl-5/Tage4/poliovirus receptor, is an immunoglobulin-like cell adhesion molecule and a member of the nectin-like family [8], [9], [10]. It is poorly expressed on normal cells, but its expression levels are upregulated on tumor cells and activated human dendritic cells (DCs) [11], [12]. Ras activation and genotoxic stress have been shown to induce CD155 expression [13], [14]. However, it is not known how CD155 expression is regulated on APCs. CD155 binds to several receptors including leukocyte adhesion-molecule DNAM-1/CD226, CD96/Tactile and TIGIT/VSTM/WUCAM [15], [16], [17]. DNAM-1 is expressed on most immune cells [18] and is upregulated on activated T_h_1 cells and T_h_2 cells [19], [20]. In contrast, CD96 and TIGIT have a more restrictive expression pattern. CD96 is expressed by NK cells, T cells and activated B cells [21]. TIGIT is absent from naïve T cells, but is expressed on activated and memory CD4^+^ T cells and regulatory T cells [16]. Recognition of CD155 by DNAM-1 or CD96 renders tumor cells sensitive to NK cells and CD8^+^ T cell-mediated cytotoxicity [22], [23]. DNAM-1 was also shown to be important for CD8^+^ T cell activation by non-professional APCs [24]. TIGIT inhibits T cell activation by inducing secretion of IL-10 and inhibiting the expression of pro-inflammatory cytokines such as IL-12 [16], [25].

Here we show that CD155 expression is significantly induced on APCs by all tested TLR agonists. CD155 upregulation by TLR signals depended on MYD88 or TRIF-mediated NF-κB activation. In addition, IRF3, but not IRF7, modulated CD155 upregulation by TLR3 agonists. A role for CD155 in regulating the T_h_2 polarization in response to TLR agonsists was suggested by increased antigen-specific IgG2a/c isotype titers and lower levels of IL-4 and fewer IL-4^+^ and GATA-3^+^ CD4^+^ T cells in the spleen of CD155-deficient mice. Hence, blocking of CD155 may be a promising novel approach to enhance the efficacy of vaccines that require strong T_h_1 responses.

## Results

### TLR Agonists Upregulate CD155 Expression on Mouse and Human APCs

**Figure 1:**
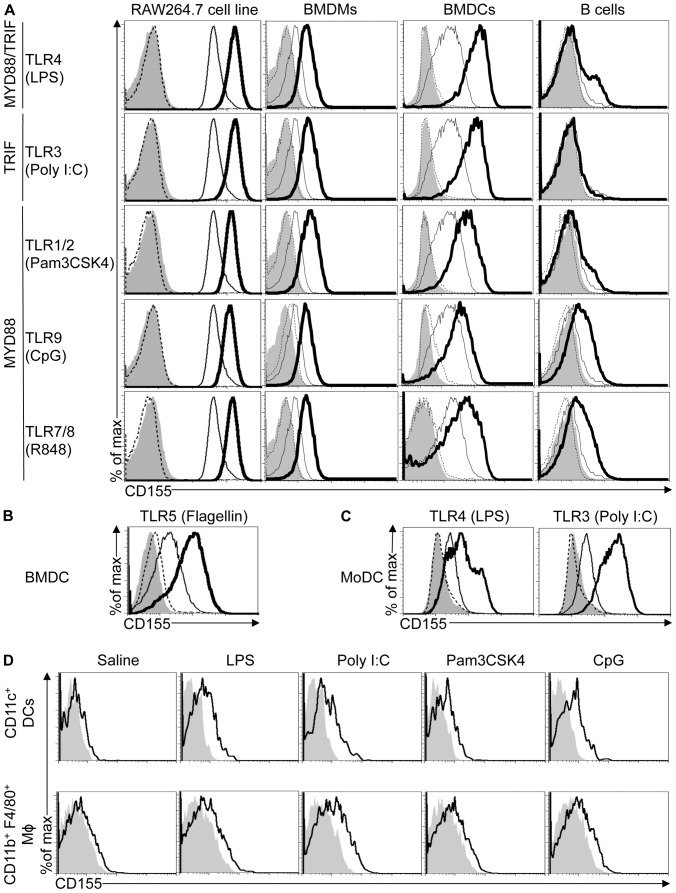
TLR agonists increase cell surface expression of CD155 on APCs. (A) RAW264.7 cells, mouse BMDMs, BMDCs and purified B cells were treated with water (thin lines) or 1 µg/ml LPS (TLR4 ligand), 25 µg/ml Poly I:C (TLR3 ligand), 40 ng/ml Pam3CSK4 (TLR1/2 ligand), 1 µM ODN 1668 (TLR9 ligand) and 1 µg/ml R848 (TLR7/8 ligand) (thick lines) for 24 or 48 hrs in the case of B cells. CD155 expression on cells was analyzed by flow cytometry. Filled histogram and dashed line indicate TLR agonist and water-treated cells stained with isotype controls. Dependency on adaptors MYD88 and TRIF by different TLRs is indicated on the left. (B and C) Mouse BMDCs (B) or human DCs (C) were treated with water (thin lines), 10 µg/ml flagellin (B), 1 µg/ml LPS (C) or 25 µg/ml Poly I:C (C) (thick lines) for 48 hrs. Cells were stained with CD155-specific antibodies and expression was analyzed by flow cytometry. Filled histograms and dotted lines represent cells stained with isotype control for TLR and water treatment, respectively. BMDMs were identified as F4/80^+^ cells, BMDCs as CD11c^+^ and B cells as CD19^+^ cells. Data are representative of at least three independent experiments. (D) C57BL/6 mice were immunized i.p. with 100 µg LPS, 100 µg Poly I:C, 50 µg Pam3CSK4, 50 µg CpG ODN 1668 or saline (thick line). 18 hrs later, splenocytes were analyzed for CD155 expression by flow cytometry. Filled histograms represent cells stained with isotype control. DCs were identified as CD11c^+^ cells and macrophages as CD11b^+^ and F4/80^+^ cells. Data are representative of two independent experiments.

To test if TLR agonists induce CD155 expression on different APCs, we treated the TLR-responsive murine macrophage-like cell line RAW264.7 with agonists for various TLRs [26]. CD155 expression was upregulated by all tested TLR agonists with the exception of flagellin, a TLR5 agonist **(** **Figure 1A** **)**. Flagellin-treated RAW264.7 cells also failed to secrete IL-6, a cytokine produced in response to TLR5 activation suggesting that RAW267.4 cells are unresponsive to TLR5 stimulation in agreement with earlier reports (data not shown) [27].

To study the effect of TLR agonists on CD155 expression in primary mouse APCs such as macrophages, DCs and B cells, we established bone marrow-derived macrophages (BMDM), bone marrow-derived DCs (BMDC) and splenic B cell cultures. Similar to RAW264.7 cells, primary BMDMs and BMDCs upregulated CD155 expression in response to all tested TLR agonists. BMDCs also upregulated CD155 expression in presence of flagellin **(** **Figures 1A and 1B** **)**. In contrast to BMDMs and BMDCs, CD155 expression was only induced by TLR7, 8 and 9 agonists in B cells, despite the ability of all tested TLR agonists to activate B cells as indicated by the upregulation of the TLR responsive gene CD40 (data not shown). Upregulation of CD155 by TLR agonists was delayed in B cells when compared to BMDMs and BMDCs **(Figure S1)**. In summary our data show that various TLR agonists can induce CD155 expression in APCs, while CD155 upregulation in B cells is restricted to MYD88-dependent TLRs expressed in the endosome. We observed no upregulation of CD155 in non-hematopoietic cells in response to different TLR agonists (data not shown).

Previous studies suggested that CD155 expression is enhanced on activated human DCs [12]. To study if TLR agonists induce CD155 expression on human DCs, we established monocyte-derived DCs (MoDCs) from human peripheral blood mononuclear cells. Treatment of MoDCs with agonists for TLR3 and TLR4, which are commonly expressed by MoDCs, induced upregulation of CD155 expression indicating that TLRs induce CD155 expression on human and mouse DCs **(** **Figure 1C** **)** [2].

To test the regulation of CD155 expression by TLR agonists *in vivo*, wild type (WT) mice were immunized with LPS, Poly I:C, Pam3CSK4, CpG or saline. Splenic DCs and macrophages of immunized mice upregulated CD155 expression in response to all tested TLRs agonists, but not saline **(** **Figure 1D** **)**.

### CD155 Upregulation Depends on the TLR Adaptors MYD88 and TRIF

**Figure 2:**
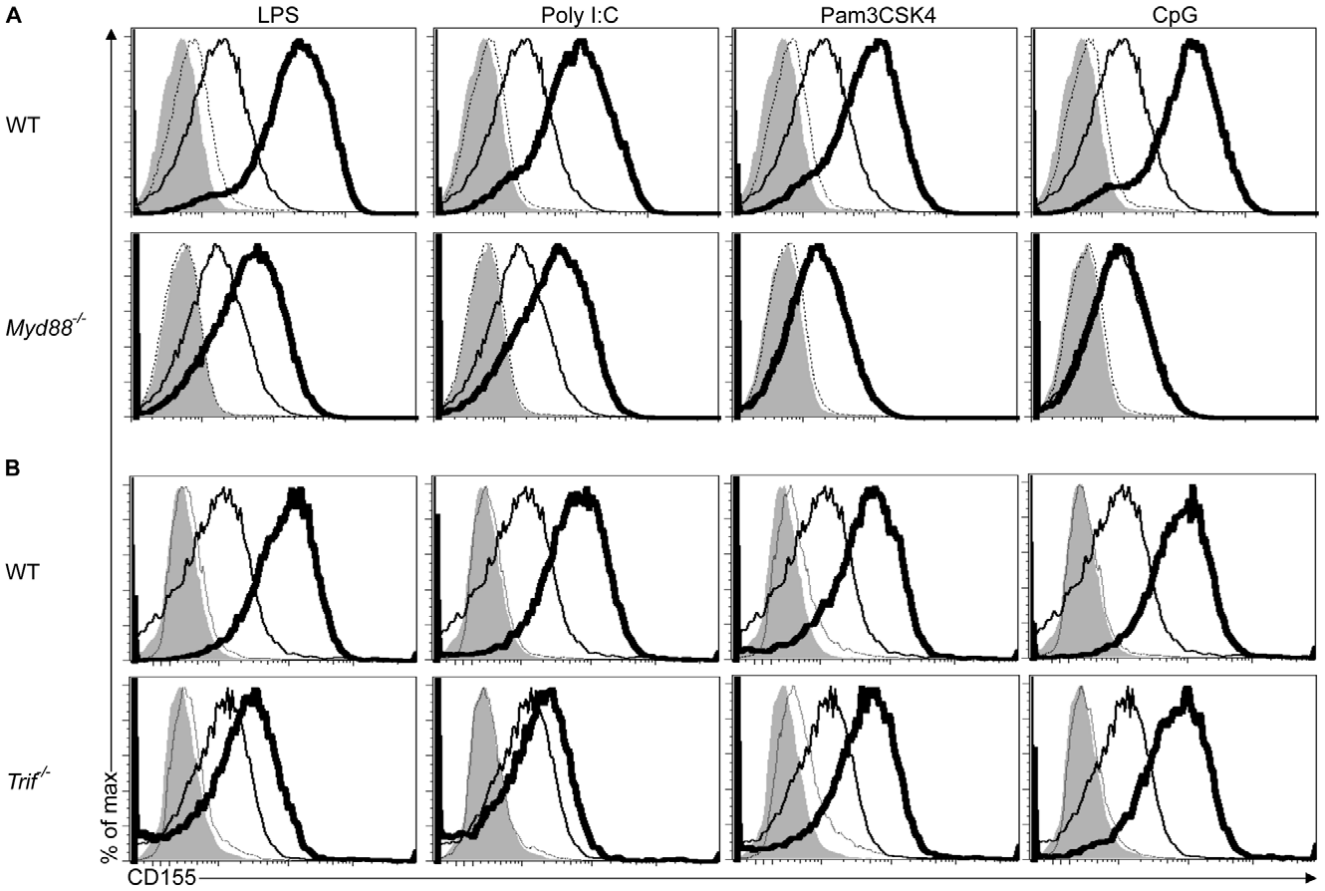
Upregulation of CD155 in response to TLR agonists depends on MYD88 and/or TRIF. (A and B) *Myd88* ^−/−^ (A), *Trif* ^−/−^ (B) and wild-type (WT) BMDCs (A and B) were treated and analyzed as indicated in figure 1. Data are representative of three independent experiments.

TLR signals are initiated by the adaptor proteins MYD88 and/or TRIF depending on the TLR [26], [28], [29], [30]. To gain better insight into the role of MYD88 and TRIF in the upregulation of CD155 expression in response to various TLR agonists, we treated *Myd88-* and *Trif*-deficient BMDCs with different TLR agonists. Induction of CD155 expression in response to TLR agonists that specifically activate MYD88, such as Pam3CSK4 and CpG was abrogated in *Myd88* ^−/−^, but not *Trif* ^−/−^ BMDCs **(** **Figures 2A and S** **2A)**. Similarly, agonists such as Poly I:C that signal exclusively through TRIF failed to upregulate CD155 expression in *Trif* ^−/−^, but not *Myd88* ^−/−^ BMDCs **(** **Figures 2B and S** **2B)**. Stimulation of *Myd88* ^−/−^ or *Trif* ^−/−^ BMDCs with LPS, a TLR4 agonist that activates both MYD88- and TRIF-dependent pathways, resulted in impaired CD155 induction when compared to WT BMDCs **(** **Figures 2** and **S2)**. These data support the conclusion that MYD88- or TRIF-initiated signals are sufficient to induce CD155 expression.

### TLR Agonists-induced CD155 Expression Depends on NF-κB

**Figure 3:**
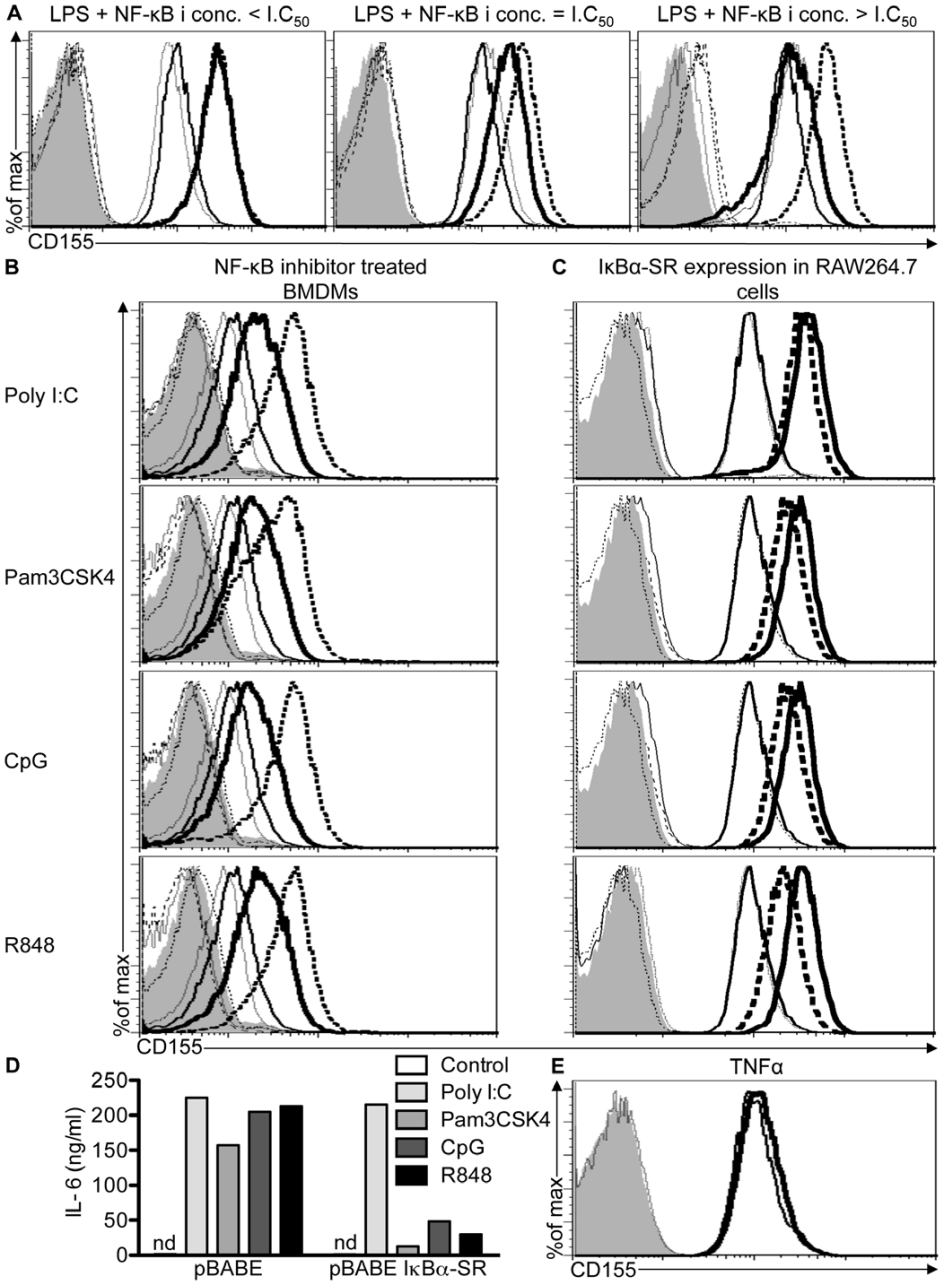
Upregulation of CD155 in response to TLR agonists depends on NF-κB. (A and B) RAW264.7 cells (A) or BMDMs (B) were pretreated with DMSO or 4 µM of the NF-κB inhibitor BMS-345541 for 1 hr followed by water or the indicated TLR agonists for 18 hrs. DMSO + water (thin line), DMSO + TLR agonist (dashed line), NF-κB inhibitor + water (dotted line) and NF-κB inhibitor + TLR agonist-treated (thick line) cells were stained with CD155-specific antibodies and analyzed by flow cytometry. DMSO + water (filled histograms), DMSO + TLR agonist (thin dotted lines), NF-κB inhibitor + water (thin dashed line) and NF-κB inhibitor + TLR agonist (dotted-dashed line) were also stained with isotype controls. Data are representative of three independent experiments. (C) RAW264.7 cells were transduced with control plasmid or plasmid encoding the IκBα super repressor (IκBα-SR) mutant. IκBα-SR transduced cells treated with water (dotted line) or TLR agonists (dashed line). Similarly, control plasmid transduced cells treated with water (thin line) or the indicated TLR agonists (thick line). All cells were stained with CD155-specific antibodies and analyzed by flow cytometry. Isotype control stainings of IκBα-SR transduced cells treated with water (thin dashed line) or TLR agonists (dotted-dashed line) and empty vector transduced cells treated with water (filled histograms) or TLR agonists (thin dotted line) are indicated. (D) IL-6 amounts in culture supernatant of cells described in figure 3C were determined by ELISA. Data are representative of two independent experiments. (E) CD155 expression on RAW264.7 cells treated with 20 ng/ml TNFα (thick line) or medium (thin line) for 18 hrs. Filled histograms and dashed line show control and TNFα-treated cells stained with isotype control. Data are representative of three independent experiments.

Activation of MYD88 and TRIF by TLR agonists leads to the activation of NF-κB [28]. To test the role of NF-κB in regulating CD155 induction, RAW264.7 cells were treated with the NF-κB inhibitor, BMS-345541, prior to stimulation with LPS [31]. Pretreatment of RAW264.7 cells with BMS-345541 at concentrations equal or higher than the published IC_50_ inhibited CD155 upregulation in response to LPS stimulation **(** **Figures 3A, S** **3A** and **S3B)**. Similar to RAW264.7 cells, pretreatment of BMDMs with BMS-345541 impaired CD155 upregulation in response to various TLR agonists suggesting that MYD88- and TRIF-induced CD155 expression critically depended on NF-κB **(** **Figures 3B and S** **3C)**. Furthermore, induction of CD155 and IL-6 expression was impaired in R848-stimulated RAW264.7 cells overexpressing the IκBα-super repressor when compared to cells expressing a control plasmid **(** **Figures 3C and 3D** **)**. IκBα-super repressor expressing RAW264.7 cells also showed a modest reduction in CD155 upregulation in response to Pam3CSK4 and CpG stimulation when compared to cells expressing the control plasmid.

To address the question if NF-κB activation was sufficient to induce CD155 expression, we treated RAW264.7 cells with TNFα, a well-characterized activator of NF-κB [32]. Although TNFα treatment induced the upregulation of CD40, a known NF-κB target gene, it failed to upregulate CD155 expression **(** **Figures 3E and S** **3D)** suggesting that NF-κB is necessary, but not sufficient to induce CD155 expression in response to TLR agonists.

### CD155 Upregulation in Response to TLR Agonists does not Depend on MAPKs

**Figure 4:**
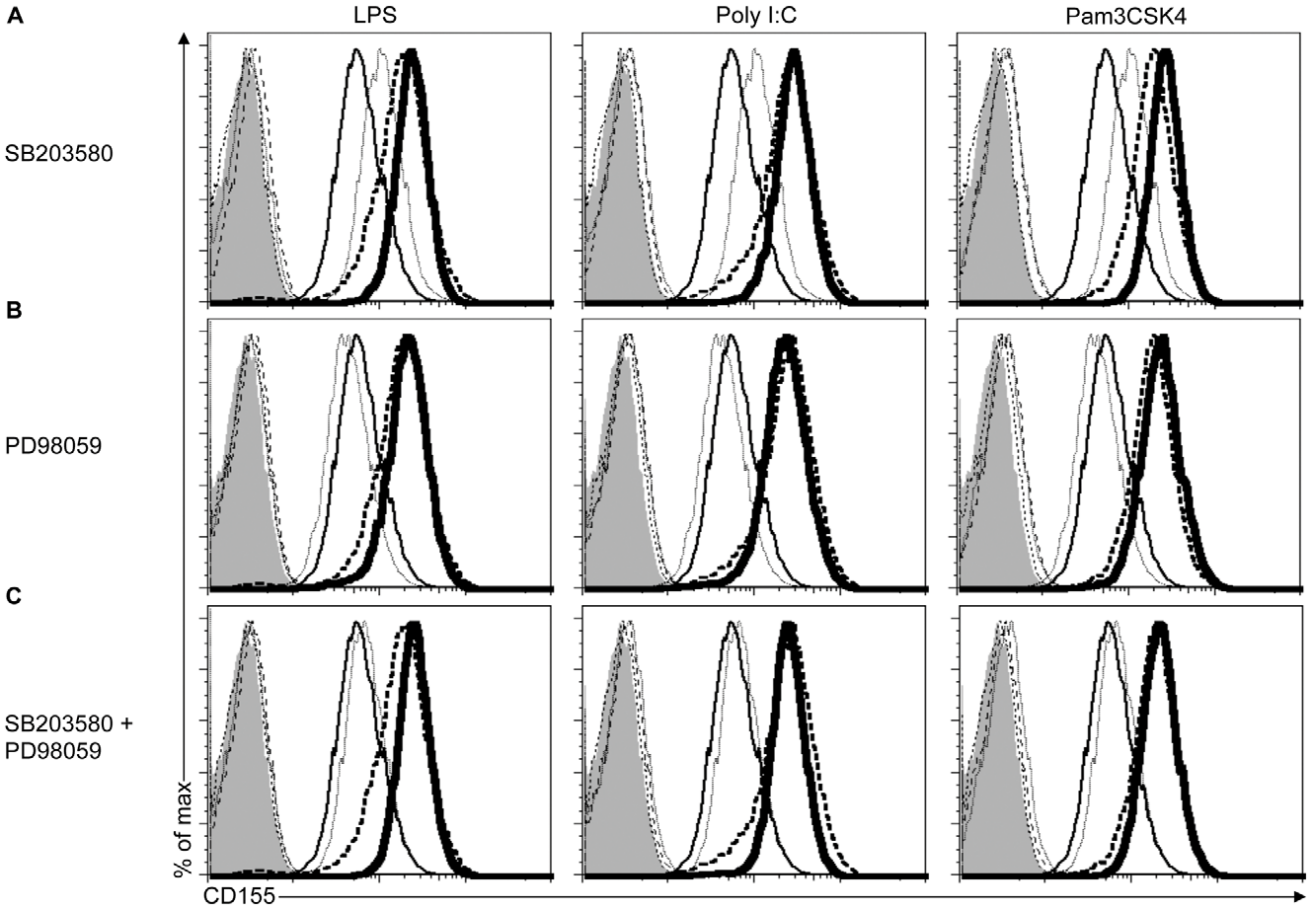
Upregulation of CD155 in response to TLR agonists is independent of MAPKs activation. (A-C) RAW264.7 cells were pretreated with DMSO (A to C), 20 µM of the p38 MAPK inhibitor SB203580 (A), 20 µM of the p42/44 MAPK inhibitor PD98059 (B) or 20 µM of SB203580+ PD98059 (C) for 1 hr followed by water or the indicated TLR agonists for 18 hrs. Cells treated with DMSO + water (thin line), DMSO + TLR agonist (dashed line), MAPK inhibitor + water (dotted line) and MAPK inhibitor + TLR agonist (thick line) were stained with CD155-specific antibodies and analyzed by flow cytometry. As a control, DMSO + water (filled histograms), DMSO + TLR agonists (thin dotted line), MAPK inhibitor + water (thin dashed line) and MAPK inhibitor +TLR agonist (dotted-dashed line)-treated cells were stained with isotype control. Data are representative of three independent experiments.

TLR agonists also activate MAPK signaling pathways [1]. We therefore tested the contribution of p38 MAPK in regulating CD155 expression. Chemical inhibition of p38 MAPK activity by SB203580 did not impair the upregulation of CD155 expression on RAW264.7 cells by LPS, Poly I:C or Pam3CSK4 **(** **Figure 4A** **)**. Similarly, inhibition of p42/p44 MAPKs by PD98059 or simultaneous blocking of p38 and p42/44 MAPKs did not affect the ability of RAW264.7 cells to induce CD155 expression **(** **Figures 4B and 4C** **)**, but inhibited LPS-induced phosphorylation of p38 and p42/44 MAPK (data not shown). Overall our data indicate that MAPKs are not required for induction of CD155 expression by TLRs.

### IRF3 Modulates TLR3-mediated Induction of CD155 Expression

**Figure 5:**
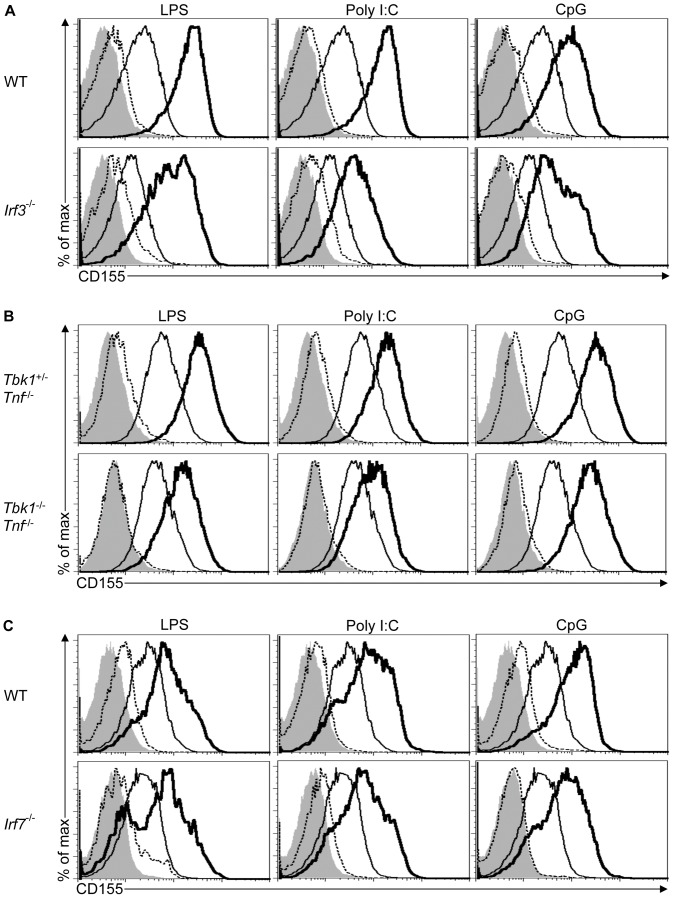
IRF3 modulates CD155 upregulation in response to TLR3 agonists. (A-C) *Irf3* **^−/−^** (A), *Tbk1* ^+**/−**^;*Tnf* **^−/−^**, *Tbk1* **^−/−^**;*Tnf* **^−/−^** (B), *Irf7^−/−^* (C) and WT BMDCs (A and C) were treated and analyzed as outlined in figure 1. Data are representative of two independent experiments.

The transcription factors IRF3 and IRF7 are important downstream regulators of the TRIF and MYD88 signaling pathways [33]. IRF3 is activated by TRIF-mediated signals in response to TLR3 and TLR4 agonists while recruitment of MYD88 by TLR7, 8, and 9 leads to the activation of IRF7 [34]. To study the role of IRF3 in CD155 expression, we analyzed the ability of *Irf3* ^−/−^ BMDCs to upregulate CD155 expression in response to Poly I:C and LPS. CD155 upregulation was impaired in *Irf3* ^−/−^ BMDCs in response to Poly I:C while upregulation in response to LPS was not affected when compared to WT BMDCs, suggesting that IRF3 is not essential for CD155 upregulation by TLR agonists that also activate MYD88 **(** **Figures 5A and S** **4A)**. IRF3 is phosphorylated and activated by the TANK-binding kinase 1 (TBK1) and/or IKK-related kinase epsilon (IKKε) in response to Poly I:C [35], [36]. To investigate the role of TBK1, we used *Tbk1* ^−/−^ BMDCs on a *Tnf* ^−/−^ background as the lethal phenotype of *Tbk1* ^−/−^ mice can be rescued by the absence of TNF [37]. Lack of TBK1 had no effect on the ability of BMDCs to upregulate CD155 expression in response to LPS, Poly I:C or the MYD88-dependent TLR agonist CpG indicating that IKKε may function redundantly with TBK1 in the activation of IRF3 and induction of CD155 expression **(** **Figures 5B and S** **4B)**.

To test the contribution of IRF7 in CD155 upregulation by TLR agonists, we treated *Irf7* ^−/−^ BMDCs with different TLR agonists. CD155 upregulation was not impaired in *Irf7* ^−/−^ BMDCs in response to TLR agonists that recruit MYD88 and/or TRIF indicating that IRF7 was not critical for CD155 upregulation **(** **Figures 5C and S** **4C)**.

### TLR Agonists Increase CD155 mRNA Levels in a NF-κB-dependent Manner

**Figure 6:**
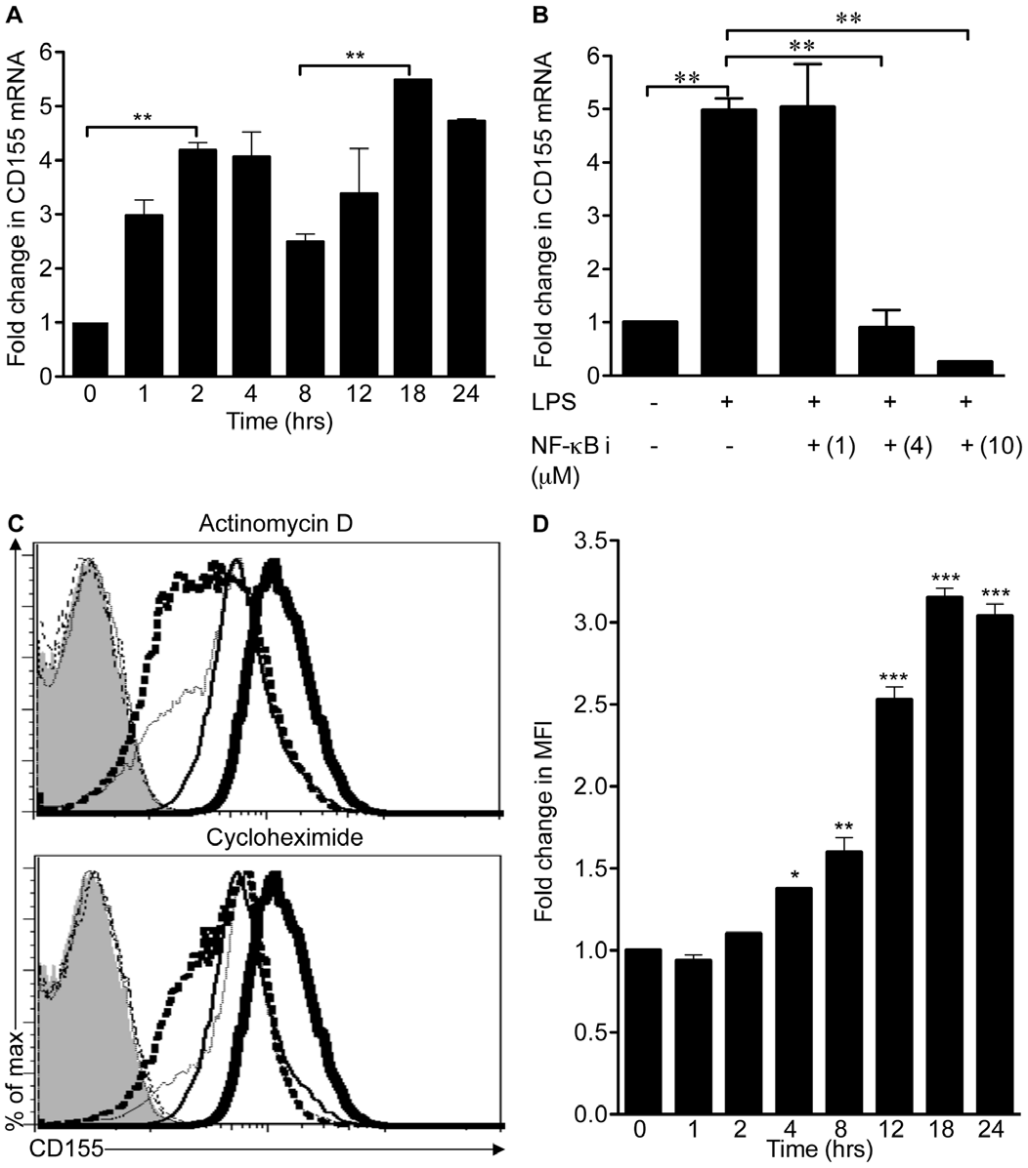
CD155 induction by TLR agonists requires *de novo* transcription and translation. (A) RAW264.7 cells were treated with 1 µg/ml LPS for the indicated time periods. Relative CD155 mRNA expression levels were analyzed by quantitative real-time PCR. (B) RAW264.7 cells were treated with DMSO or the NF-κB inhibitor, BMS-345541 at the indicated concentrations for 1 hr followed by treatment with 1 µg/ml LPS for an additional 4 hrs. Expression of CD155 was analyzed by real-time PCR. (C) RAW264.7 cells were treated with DMSO (thin line), 5 µg/ml actinomycin D (thin dotted line in the upper panel), 50 µg/ml cycloheximide (thin dotted line in the lower panel), LPS (thick lines), the combination of actinomycin D and LPS (thick dashed line in the upper panel) or cycloheximide and LPS (thick dashed line in lower panel). Filled histograms represent isotype control stainings of cells treated with DMSO, thin dotted lines show actinomycin- or cycloheximide-treated cells and thin dashed lines indicate LPS and actinomycin- or cycloheximide-treated cells. (D) LPS-treated RAW264.7 cells shown in (A) were also analyzed for expression of CD155 by flow cytometry. For A-D results were obtained from three independent experiments. Results shown in (A, B and D) were normalized to untreated cells and data is presented as mean fold change ± SEM. Mean fold change between groups was compared using one-way ANOVA followed by Bonferroni post test. * *p*<0.05, ** *p*<0.01, *** *p*<0.005.

Our data supported the possibility that CD155 expression is directly regulated by TLR-mediated activation of the transcription factor NF-κB. To gain better insight into potential transcriptional regulation of CD155 in response to TLR agonists, we measured mRNA levels by quantitative real-time RT-PCR upon LPS stimulation **(** **Figure 6A** **)**. The induction of CD155 mRNA levels by LPS followed the biphasic expression pattern typical of NF-κB target genes [1]. The increase of CD155 mRNA levels in response to LPS stimulation was significantly impaired by pretreating cells with the NF-κB inhibitor BMS-345541 at concentrations above the published IC_50_ **(** **Figure 6B** **)**. Furthermore, pretreatment of RAW264.7 cells with the transcriptional inhibitor actinomycin D abrogated upregulation **(** **Figure 6C** **)**. In contrast to the mRNA level, CD155 protein levels steadily increased after 3 to 5 hours of TLR agonist treatment and CD155 reached maximal expression after 18 hours of treatment **(** **Figures 6D and S** **5)**. Pretreatment of RAW264.7 cells with the protein synthesis inhibitor cycloheximide prevented induction of CD155 expression in response to LPS **(** **Figure 6C** **)**. In summary our data suggest that TLR agonists regulate CD155 expression at the transcriptional and possibly posttranscriptional level.

### CpG-induced CD155 Expression Modulates Antigen-specific IgG2a/c Titers

**Figure 7:**
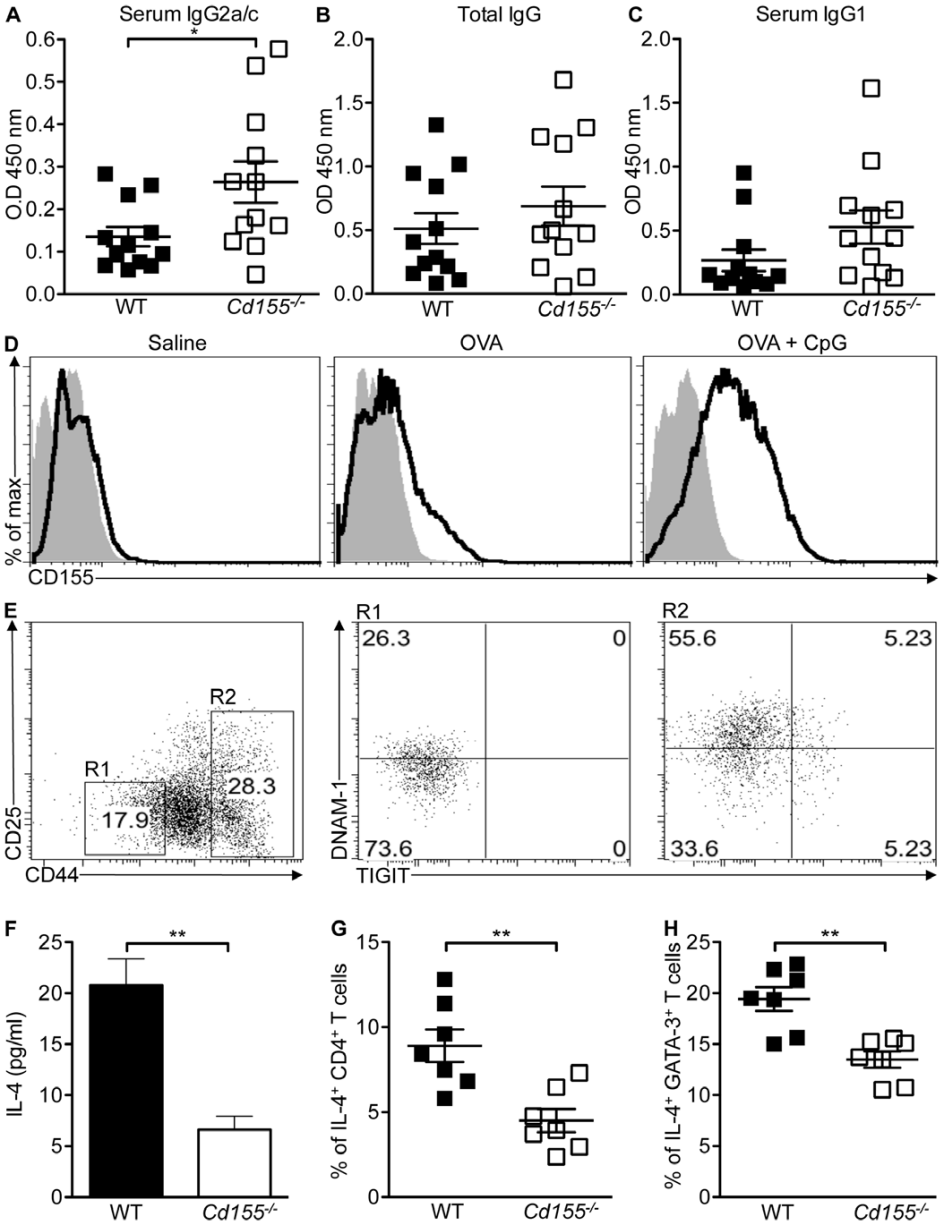
Increase of OVA-specific IgG2a/c antibody titer in *Cd155^−/−^* mice immunized with OVA and CpG. (A-D and F-H) *Cd155^−/−^* mice (n  = 12) and WT littermate (n  = 12) were immunized i.p. with 20 µg OVA and 50 µg ODN 1668, 20 µg OVA or saline alone. One week later the immunization was repeated. 21 days after first immunization, serum levels of OVA-specific IgG2a/c (A), total IgG (B) and IgG1 (C) antibodies in OVA- and CpG-immunized *Cd155^−/−^* mice (open squares) and WT littermates (closed squares) were determined by indirect ELISA at a 1∶100,000 serum dilution. Combined data of three independent experiments are shown. Groups shown in (A-C) were compared using Student’s t-test. Means ± SEM. are indicated, * *p*<0.05. (D) Splenic B cells (CD19^+^ cells) of saline, OVA and OVA + CpG ODN 1668-injected WT mice were analyzed for CD155 expression (thick line) by flow cytometry. Filled histogram shows B cells stained with isotype control. (E) Splenocytes of WT mice (n  = 3) were stained for CD3, CD4, CD44, CD25, TIGIT and DNAM-1 expression. Dot blots show TIGIT and DNAM-1 expression in splenocytes electronically gated on CD3^+^CD4^+^CD25^−^CD44^low^ (R1, naïve phenotype, middle panel) or CD3^+^CD4^+^CD44^high^ (R2, effector/memory phenotype, right panel) cells. Left panel illustrates CD25 and CD44 gating strategy used for R1 and R2. (F) 5×10^6^ splenocytes of mice injected with OVA and CpG were cultured for 48 hrs, after which the culture supernatant was analyzed for IL-4 levels by ELISA. Data are representative of two independent experiments. (G and H) CD3^+^CD4^+^ splenocytes of OVA- and CpG-immunized WT and *Cd155^−/−^* mice (n  = 7) were analyzed for intracellular IL-4 (G) and GATA-3 (H) expression by flow cytometry. Data presented in (F-G) were analyzed using Student’s t-test and means ± SEM are shown. ** *p*<0.01, *ns* indicates not significant.

TLRs regulate the generation of the adaptive immune responses by modulating the induction of accessory signals such as costimulatory molecules and cytokines [2]. The TLR9 agonist CpG has been successfully used as an adjuvant to boost immunity against influenza virus, measles virus and hepatitis B surface antigen [38]. The ability of CpG to enhance the immune response critically depended on MYD88 signaling in B cells [39], [40], [41]. Furthermore, CD155 expression on B cells was shown to be important for the activation of CD8^+^ T cells [42]. To test the role of CD155 in the adjuvant effect of CpG, *Cd155* ^−/−^ and WT mice were immunized with CpG together with OVA or OVA alone. 21 days after immunization, OVA-specific serum IgG levels were measured by ELISA. *Cd155* ^−/−^ mice immunized with CpG and OVA produced significantly higher titers of OVA-specific IgG2a/c antibodies than WT mice **(** **Figures 7A and S** **7)**. IgG2a/c titers were analyzed as CD155-deficient mice were on a mixed genetic background and some mouse strains express IgG2c instead of IgG2a [43], [44]. We observed no significant differences in the levels of OVA-specific total IgG or other IgG isotypes in *Cd155* ^−/−^ mice when compared to WT mice **(** **Figures 7B, 7C, S** **6A, S6B** and **S7)**. In further support for a role of CD155 in CpG-mediated adjuvant effects, splenic B cells of OVA and CpG immunized mice expressed significantly higher amounts of CD155 when compared to OVA only-injected mice **(** **Figure 7D** **)**. IgG2a/c isotype switching is associated with T_h_1 responses. Costimulatory molecules have been shown to promote T_h_2 immunity and to test the possibility that CD155 modulated IgG2a/c titers through the polarization of naïve CD4^+^ T cells, we determined if the CD155-binding receptors DNAM-1 and TIGIT are expressed on naïve CD4^+^ T cells of WT and *Cd155^−/−^* mice. CD4^+^ T cells with a naïve phenotype (CD4^+^CD25^−^CD44^low^) expressed DNAM-1, but not TIGIT. DNAM-1 expression was significantly higher on CD4^+^ T cells of *Cd155^−/−^* mice when compared to WT mice **(** **Figures 7E, S** **6C** and **S6D)**. *Cd155* ^−/−^ splenocytes of OVA and CpG-immunized mice secreted significantly lower levels of IL-4, a cytokine shown to drive T_h_2 differentiation, when compared to WT splenocytes **(** **Figure 7F** **)** [45]. Furthermore, spleens of OVA and CpG immunized *Cd155^−/−^* mice harbored significantly less IL-4 and GATA-3 expressing CD4^+^ T cells when compared to WT mice **(** **Figures 7G and 7H** **)**. In contrast, the percentage of IFN-γ and T-Bet expressing CD4^+^ T cells was similar between *Cd155^−/−^* and WT mice **(Figures S6E, S6F** and **S6G)**. In summary, our data suggest that CpG-induced CD155 expression suppresses IgG2a/c isotype switching by regulating T_h_2 polarization of naïve CD4^+^ T cells.

## Discussion

Our study shows that CD155 expression is upregulated in response to various TLR agonists on APCs. Using APCs deficient in MYD88 or TRIF, we found that MYD88 and TRIF-initiated pathways were sufficient to induce CD155 expression and when activated together exhibited an additive effect on CD155 upregulation. Chemical and genetic inhibition of NF-κB supported the conclusion that NF-κB was critical for MYD88/TRIF-mediated CD155 upregulation. In contrast, IRF3 was not essential for TLR-mediated CD155 induction, but modulated CD155 expression in response to TLR3 agonists that activate TRIF. Our findings are in accordance with Andersen et al. who recently classified CD155 as an IRF3 augmented gene [46]. Other IRFs may contribute to CD155 induction as several TLR agonists activate IRF5 and IRF7, although our data do not support a role for IRF7 [47]. Coregulation of genes by NF-κB and IRFs has been found for the transcriptional regulation of other cell adhesion proteins such as ICAM-1 and VCAM-1 [48]. Preliminary analysis of the CD155 promotor indicated potential NF-κB and IRF transcription factor binding sites in close proximity to the transcription start site of CD155 (data not shown). It has previously been reported that CD155 contains AP-1 transcription factor binding sites in its promoter [14]. However, chemical inhibition of MAPK pathways that lead to AP-1 activation, did not abrogate CD155 upregulation suggesting that AP-1 is not required for CD155 induction in response to TLR agonists. Future studies will be needed to address the role of these potential binding sites and transcription factors in the regulation of CD155 expression by TLR activation.

TLRs are essential for the generation of a robust adaptive immune responses by regulating the generation of T_h_ cells and effector cytokines [2]. Upon stimulation of naïve T_h_ cells by cognate antigen presented on APCs, T_h_ cells differentiate into different subsets. Two important T_h_ cells subsets are T_h_1 cells, which produce IFN-γ and promote immunity against intracellular pathogens and T_h_2 cells, which secrete IL-4, IL-5 and IL-13 and generate immunity against extracelluar parasites. TLRs are critically involved in the early T_h_ cell fate decision. MYD88-deficient mice were found to be impaired in the activation of antigen-specific T_h_1, but not T_h_2 cell responses [49]. Furthermore, stimulation of APCs with the MYD88 activating TLR9 agonist CpG induced the secretion of T_h_1-associated cytokines and the expression of co-stimulatory molecules on APCs [50], [51]. Administration of CpG and antigen directs antibody production by murine B cells to T_h_1-associated immunoglobulin isotypes, such as IgG2 and IgG3, while suppressing IgG1 and IgE-associated T_h_2 isotypes [52]. In contrast, signals by CD28 and other co-stimulatory molecules preferentially promote T_h_2 differentiation [53], [54]. Interestingly, CD155 plays an important role in T_h_1-type cellular immunity by promoting IFN-γ production by NK cells and CD8^+^ T cell activation by non-professional APCs such as B cells [12], [42]. DNAM-1, a receptor that binds CD155, was reported to be upregulated on T_h_1 cells and to regulate their effector functions [19]. We found that *Cd155^−/−^* mice immunized with CpG and OVA produced higher titers of T_h_1-associated isotypes IgG2a/c in comparison to WT littermates suggesting that CpG-induced CD155 expression suppresses T_h_1 cell differentiation. Consistent with this possibility, splenocytes of immunized *Cd155* ^−/−^ mice produced significantly less IL-4. Furthermore, a lower percentage of CD4^+^ T cells expressed intracellular IL-4 and the T_h_2 promoting transcription factor GATA-3. As IFN-γ and IL-12p70 levels were similar between WT and *Cd155^−/−^* splenocytes our data indicate that CD155 does not regulate differentiation of T_h_2 cells, but not T_h_1 cells **(Figures S6E** and **S6H)**. It is unlikely that CD155 mediates these effects by binding to TIGIT as CD4^+^ T cells with a naïve phenotype did not express detectable levels of TIGIT at the cells surface and TIGIT is expressed at similar levels on T_h_1 and T_h_2 subsets (**Figures 7E and S** **6D**) [16]. Furthermore, our data do not suport a role for CpG-induced CD155 expression in the differentiation of regulatory T cells as splenocytes of immunized *Cd155* ^−/−^ and WT mice with OVA and CpG induced similar amounts of IL-10, a cytokine produced by regulatory T cells **(Figure S6I)**. In summary, our data suggest that CpG-induced expression of CD155 on B cells and possibly other APCs modulates the humoral immune response by polarizing naïve CD4^+^ T cells towards a T_h_2 phenotype.

## Methods

### Ethics Statement

Mice were housed and bred in pathogen free conditions in strict compliance with the Institutional Animal Care and Use Committee (IACUC) guidelines at the National University of Singapore, in accordance with the National Advisory Committee for Laboratory Animal Research (NACLAR) Guidelines (Guidelines on the Care and Use of Animals for Scientific Purposes). The protocol was approved by Institutional Animal Care and Use Committee of the National University of Singapore (Protocol Number: 041/08) and steps were taken to minimize suffering. All research involving human samples was conducted according to the principles expressed in the Declaration of Helsinki and was approved by the National University of Singapore Institutional Review Board (NUS-IRB), Singapore. Peripheral blood samples were obtained with written informed consent from human volunteers.

### Mice

C57BL/6 mice were purchased from the Centre for Animal Resources at the National University of Singapore. *Irf3^−/−^* mice on a C57BL/6 background were purchased from the RIKEN Bioresource Centre (Japan). *Irf7^−/−^* mice on a C57BL/6 background were generously provided by Dr. F. Ginhoux (Singapore Immunology Network, Singapore) [55]. The *Cd155^−/−^* mice were generated as described in Abe et al. [56]. The genetic background of the *Cd155^−/−^* mice was 129/Sv (50%), C57BL/6 (25%) and DBA (25%). Offsprings of *Cd155^−/−^* mice×C57BL/6 mice breedings were used for all experiments.

### Cell Culture

The mouse monocytic leukemic macrophage cell line RAW264.7 (TIB-71, ATCC, USA) was cultured in RPMI 1640 medium (Invitrogen, Singapore) supplemented with 5% heat inactivated FCS (Invitrogen, Singapore), 20 mM HEPES (HyClone, USA), 1.4 µM L-glutamine (Sigma, Singapore), 20 U/mL penicillin-streptomycin (Invitrogen, Singapore), 5.2 mg/ml gentamicin sulphate (Invitrogen, Singapore) and 1.82****µl/ml β-mercaptoethanol (Sigma, Singapore). Bone marrow from *Myd88* ***^−/−^*** and *Trif* ***^−/−^*** mice was generously provided by Dr. S. Biswas (Singapore Immunology Network, Singapore) [57]–[58]. *Tbk1* ***^−/−^***;*Tnf* ***^−/−^*** and *Tbk1* ***^+/−^***;*Tnf* ***^−/−^*** bone marrow was kindly provided by Dr. K. Ishii (Osaka University, Japan) [59]. *Tbk1* ***^−/−^***;*Tnf* ***^−/−^***, *Tbk1* ***^+/−^***;*Tnf* ***^−/−^***, *Myd88* ***^−/^,*** *Trif^−/−^*, *Irf3* ***^−/−^*** and *Irf7^−/−^* BMDCs were generated as described by Inaba et al [60]. Bone marrow derived macrophages (BMDMs) were generated and cultured as described before [61]. Human PBMCs of healthy volunteers were isolated by Ficoll-Hypaque (Sigma, Singapore) gradient centrifugation. Monocytes were purified using the MACS CD14 isolation kit (Miltenyi Biotech, Singapore) and were subsequently cultured in 6 well dishes (1.5–2×10^6^ cells/ml) in the presence of 800 U/ml of GM-CSF and 500 U/ml of IL-4 (eBioscience, USA) to generate monocyte-derived dendritic cells (MoDCs). Mouse B cells were purified from spleen using the mouse EasySep CD19 positive selection kit (STEMCELL Technologies, Singapore). Splenic B cells were cultured in 10% FCS supplemented RPMI 1640 medium (Invitrogen, Singapore). All cells were grown at 37°C in a humidified 5% CO_2_ incubator (Thermo Scientific, Singapore).

### Treatments

All TLR ligands were obtained from Invivogen (USA). Human MoDCs were treated for 48 hrs as follows: 1 µg/ml LPS and 25 µg/ml Poly I:C. RAW264.7 cells, mouse BMDCs, BMDMs and B cells were treated with 1 µg/ml LPS, 25 µg/ml Poly I:C, 40 ng/ml Pam3CSK4, 1 µM CPG ODN 1668 and 1 µg/ml R848 for 24 hrs or 48 hrs in the case of B cells. BMDCs were stimulated with 100 ng/ml flagellin derived from S. *typhimurium* for 24 hrs. RAW264.7 cells were treated with 20 ng/ml of TNFα (eBioscience, USA) for 24 hrs.

For analysis of CD155 expression *in vivo*, C57BL/6 mice were immunized with saline, 100 µg LPS, 100 µg Poly I:C, 50 µg Pam3CSK4 and 50 µg CpG ODN 1668 intraperitoneally (i.p.). 18 hrs later splenocytes were harvested and stained for CD155 expression.

RAW264.7 cells transduced with IκBα dominant-negative mutant (Addgene, USA) or empty vector plasmid were treated as indicated above in 6 well dishes at a density of 2×10^5^ cells in each well. Cells were used for FACS and culture supernatants tested for IL-6 by ELISA (eBioscience, USA) according to the manufacturer’s instructions.

To analyze the kinetics of CD155 expression, 2×10^6^ RAW264.7 cells were stimulated with 1 µg/ml LPS for different times. At indicated time, cells were harvested and CD155 expression was assessed by real-time RT-PCR and flow cytometric analysis. In order to test whether CD155 upregulation requires *de novo* transcription or translation, 1×10^6^ RAW264.7 cells in a 10 cm dish were treated with 5 µg/ml actinomycin D (Sigma, Singapore) or 50 µg/ml cycloheximide (Calbiochem, Singapore) for 1 hr, followed by treatment with 1 µg/ml LPS for 5 hrs.

For pharmacological inhibition of NF-κB or MAPK signaling, 1×10^6^ BMDMs or 2×10^6^ RAW264.7 cells were seeded in 6-well plates and 10 cm dishes respectively. The next day cells were pretreated with 1 µM or 4 µM or 10 µM BMS-345541 or 20 µM SB203580 or 20 µM PD98059 (Calbiochem, Singapore) for 1 hr, followed by stimulation with TLR agonists at doses indicated above for an additional 24 hr. For real time and western blot assays of NF-κB inhibitor treated cells, the TLR ligand stimulation time was shortened to 5 hrs.

### Transduction

The pBABE-puro-IκBα (Addgene plasmid 15291) and empty pBABE-puro plasmids were obtained from Addgene [62]. Transduction of cells was performed as described before [63]. 48 hrs after transduction, cells were selected with 5 µg/ml puromycin (Sigma, Singapore) for at least 7 days.

### Flow Cytometry

PE anti-human CD155, APC anti-mouse CD155, Alexa-fluor 647 anti-mouse TIGIT, PE anti-mouse CD40, PB anti-mouse CD11c, PE anti-mouse F4/80, PB anti-mouse CD19, FITC anti-mouse CD3, PB anti-mouse CD4, APC anti-mouse CD8, PB anti-mouse CD44, FITC anti-mouse CD25, PE anti-mouse CD80 and FITC anti-mouse CD86 antibodies were purchased from eBioscience (USA). DNAM-1 and TIGIT expression on naïve T cells was studied using spleens harvested from 8 weeks old WT and *Cd155^−/−^* mice. Data was acquired using a CyAn ADP analyzer (Beckman Coulter, Singapore) and FlowJo software version 8.8.7 (TreeStar, USA). All plots show cells gated for viable cells, as determined by live-cell gating by forward scatter, side scatter, and lack of propidium iodide (Sigma, Singapore) uptake; viability of cells for all experiments was >80%.

Intracelluar IFN-γ and IL-4 staining was performed using the BD Cytofix/Cytoperm kit (BD Biosciences, Singapore) following the manufacturer’s instructions. For staining of T-Bet and GATA-3, Foxp3/Transcription Factor Staining Buffer Set (eBioscience, USA) was used. Splenocytes were harvested from OVA and CpG immunized WT and *Cd155^−/−^* mice on day 21 after immunization. 5×10^6^ splenocytes were cultured in 1 ml of medium for 24 hours. 20 ng/ml PMA and 500 ng/ml Ionomycin (Invitrogen, Singapore) was added to the culture for the last 6 hours together with 1 µl/ml each of GogliPlug and GolgiStop (BD Biosciences, Singapore). Cells were first stained with APC-Cy7 conjugated Fixable Viability dye (eBioscience, USA) followed by staining with FITC anti-mouse CD3 and PB anti-mouse CD4 antibodies. Cells were then fixed and permeabilized using the components provided in the BD Cytofix/Cytoperm kit and stained with APC anti-mouse IFN-γ (BD Biosciences, Singapore) and PE anti-mouse IL-4 (BD Biosciences, Singapore) antibodies or fixed and permeabilized using the eBioscience Foxp3/Transcription Factor Staining Buffer Set and stained with PE conjugated T-Bet (eBioscience, USA) and APC conjugated GATA-3 antibodies (eBioscience, USA). Data was acquired using a CyAn ADP analyzer (Beckman Coulter, Singapore) and analyzed using FlowJo software version 8.8.7 (TreeStar, USA).

### Real-time PCR

Total RNA was isolated using the RNeasy kit according to the manufacturer’s instructions (Qiagen, Singapore). 2 µg of total RNA was reverse transcribed using random hexamer primer and M-MLV reverse transcriptase (Promega, Singapore). Each amplification mixture (25 µl) contained 50 ng of reverse transcribed RNA, 0.8 µM forward primer, 0.8 µM reverse primer and 12.5 µl of iTaq SYBR Green Supermix with ROX (Bio-Rad Laboratories, Singapore). PCRs were performed in triplicates using the ABI PRISM 7700 Sequence Detection System from Applied Biosystems. PCR thermocycling parameters were 50°C for 2 min, 95°C for 10 min, and 40 cycles of 95°C for 15 sec and 60°C for 15 sec and 72°C for 1 min. All samples were normalized to the signal generated from the housekeeping gene *Hprt*. The following primers were used: *Hprt*-5′: tgggaggccatcacattgt; *Hprt*-3′: gcttttccagtttcactaatgaca; *Cd155*-5′: cgtgtccatctctggctatg; *Cd155*-3′ cgtgttcgtgctccagttat. Samples prepared without RNA served as negative control templates. Experiments were analyzed using GraphPad Prism software (Version 5.0a, GraphPad Software, USA).

### Western Blotting

Cells were lysed in Radio-Immunoprecipitation Assay buffer (RIPA) consisting of 50 mM Tris-HCl, pH 7.4, 150 mM NaCl, 1 mM EDTA, 1% NP-40 and 1% sodium deoxycholate (Sigma). Prior to lysis, protease inhibitor cocktail set III (Merck, Singapore) and phosphatase inhibitor cocktail set V (Merck, Singapore) were added to the lysis buffer according to the manufacturer’s instructions. 35 µg of lysate was loaded on a 10% reducing SDS PAGE gel. Lysate was transferred to a nitrocellulose membrane (Amersham, Singapore) and probed with anti-mouse IκBα antibody (Cell Signaling Technology, USA) followed by HRP-conjugated goat anti-rabbit IgG (Jackson Immunoresearch, USA). Blots were reprobed for tubulin expression using a mouse tubulin specific antibody (Sigma, Singapore) followed by HRP-conjugated goat anti-mouse IgG antibody (Jackson Immunoresearch, USA).

### OVA Immunization

20 µg ovalbumin (OVA) (Grade V, Sigma, Singapore) or 20 µg OVA and 50 µg CpG ODN 1668 (Invivogen, USA) was administered i.p. to 7 to 8 week old CD155-deficient mice and age matched littermate controls. Blood samples were taken one day before injection by facial vein bleeding. On day 21 mice were sacrificed and blood samples were obtained by cardiac puncture. Serum was analyzed for OVA-specific antibody titer using indirect ELISA [40]. Briefly, 96 well MaxiSorp plates (Nunc, Singapore) were coated overnight at 4°C with 10 µg/ml of OVA (Sigma, Singapore) dissolved in 0.1 M carbonate/bicarbonate buffer at pH 9.6. Plates were blocked with 0.05% PBS-Tween containing 2% BSA (Sigma, Singapore) and 5% goat serum (Invitrogen, Singapore) for 2 hrs at room temperature. After washing, plates were incubated with serially diluted serum samples and incubated overnight at 4°C. OVA-specific IgG, IgG1 and IgG3 levels were determined by incubating plates for 1 hr with peroxidase-conjugated goat anti-mouse IgG, Fc_γ_ fragment specific, goat anti-mouse IgG, Fc_γ_ subclass 1 specific or and goat anti-mouse IgG, Fc_γ_ subclass 3 specific antibodies (Jackson Immunoresearch, USA). To analyze IgG2a/c titers, a 1∶1 molar ratio of goat anti-mouse IgG Fc_γ_ subclass 2a specific and goat anti-mouse IgG Fc_γ,_ subclass 2c-specific antibodies was added to the plates. After 1 hr, plates were washed and incubated with 3,3′,5,5′-Tetramethylbenzidine (TMB) substrate according to the manufacturer’s instructions (eBioscience, USA). Results were analyzed using GraphPad Prism software (Version 5.0a, GraphPad Software, USA).

### Cytokine ELISA

5×10^6^ spleen cells of OVA and CpG immunized mice were cultured in 1 ml RPMI medium (Invitrogen, Singapore) for 48 hrs after which, the levels of IL-4, IFN-γ, IL-12p70 and IL-10 in the supernatant was measured by ELISA according to the manufacturer’s instructions (eBioscience, USA).

## Supporting Information

Figure S1 **B cells show a delayed kinetics of CD155 upregulation in response to TLR agonists.** B cells purified from the spleens of WT mice were treated with control (thin line) or 1 µM CpG (thick line) for the indicated times. Subsequently, cells were analyzed for CD155 expression by flow cytometry. Filled histograms and dotted lines represent control and CpG-treated cells stained with isotype control antibodies. Data are representative of three independent experiments.
(TIFF)

Figure S2 **CD155 upregulation in response to TLR agonists depends on MYD88 and TRIF.** Replicate staining of BMDCs derived from *Myd88* ^−/−^ (A) and *Trif* ^−/−^ (B) mice is shown.
(TIFF)

Figure S3 **NF-κB inhibitor** **BMS-345541 blocks TLR-induced CD155 upregulation.** (A) RAW 264.7 cells were treated with BMS-345541 for 1 hr followed by 1 µg/ml LPS for 5 hrs. NF-κB activation was detected by western blotting as a decrease in IκBα levels. Tubulin levels were used as a loading control. (B) Analysis of CD155 expression on RAW264.7 cells shown in figure 3A. (C) Analysis of CD155 expression on BMDMs shown in figure 3B. Groups shown in (B-C) were combined from three independent experiments and represent fold change of MFI ± SEM, * *p*<0.05, ** *p*<0.01, *** *p*<0.005. (D) RAW264.7 cells were treated with PBS (thin black line) or 20 ng/ml TNFα (thick black line) for 24 hrs and stained with CD40-specific antibody. Filled histograms and dotted line represent control and TNFα-treated cells stained with isotype control. Data are representative of two independent experiments.
(TIFF)

Figure S4 **Induction of CD155 expression in response to TLR agonists depends on IRF3.** Replicate staining of BMDCs derived from WT, *Irf3* ^−/−^ (A), *Tbk1* ^+/−^;*Tnf* ^−/−^, *Tbk1* ^−/−^;*Tnf* ^−/−^ (B) and *Irf7^−/−^* (C) mice.
(TIFF)

Figure S5 **CD155 protein expression increases in response to TLR stimulation.** Untreated RAW264.7 cells (thin line) or RAW264.7 cells were treated with 1 µg/ml LPS for 2 (thin dashed line), 4 (thin dotted line), 8 (thick line), 18 (thick dotted line) and 24 hrs (thick dashed line) and analyzed for CD155 expression by flow cytometry. Untreated cells stained with isotype control antibody are shown as filled histogram. Data are representative of three independent experiments.
(TIFF)

Figure S6 **(A-B) Serum levels of OVA-specific IgG2b (A) and IgG3 (B) antibodies in** ***Cd155^−/−^*** **(n  = 12) mice and WT (n  = 12) littermates injected i.p. with OVA and CpG. Titers of OVA-specific antibodies were measured and analyzed as outlined in** **figure 7** **.** Combined data of three independent experiments are shown. (C-D) Analysis of DNAM-1 (C) and TIGIT (D) expression on CD3^+^CD4^+^CD25^−^CD44^low^ and CD3^+^CD4^+^ CD44^high^ CD4^+^ T cells from spleens of WT and *Cd155^−/−^* mice. Groups were compared using one-way ANOVA followed by Bonferroni post test and are expressed as means ± SEM, * *p*<0.05, ** *p*<0.01. (E) 5×10^6^ splenocytes of WT and *Cd155^−/−^* mice injected with OVA and CpG were cultured for 48 hrs, after which the culture supernatant was analyzed for IFN-γ levels by ELISA. Data are representative of two independent experiments. (F and G) Splenocytes of OVA and CpG-immunized WT and *Cd155^−/−^* mice (n = 7) were analyzed for intracellular IFN-γ (F) and T-Bet (G) expression by flow cytometry. (H and I) Cell culture supernatants from cultured spleen cells of OVA and CpG-immunized WT and *Cd155^−/−^* mice were analyzed for amounts of IL-12p70 (H) and IL-10 (I). Results shown in (E-I) were compared using Student’s t-test and are expressed as means ± SEM. *ns* indicates not significant.
(TIFF)

Figure S7 **Increased IgG2a/c titers in OVA and CpG-immunized** ***Cd155^−/−^*** **mice when compared to WT mice.** Results obtained in figure 7 (A) – (C) and Figure S6 (A) – (B) are shown as endpoint dilutions. Titers were calculated as the reciprocal of the last serum dilution that resulted in an OD at 490 nm above that of double the corresponding value obtained with the pre-immune serum. Data were compared using the Mann Whitney U test. * *p*<0.05.
(TIFF)
