# Supplementary figures and images for: Toll-Like Receptor Ligands Induce Expression of the Costimulatory Molecule CD155 on Antigen-Presenting Cells
Source: PLoS One. 2013 Jan 17;8(1):e54406. doi: 10.1371/journal.pone.0054406 (PMC3547938; doi:10.1371/journal.pone.0054406)

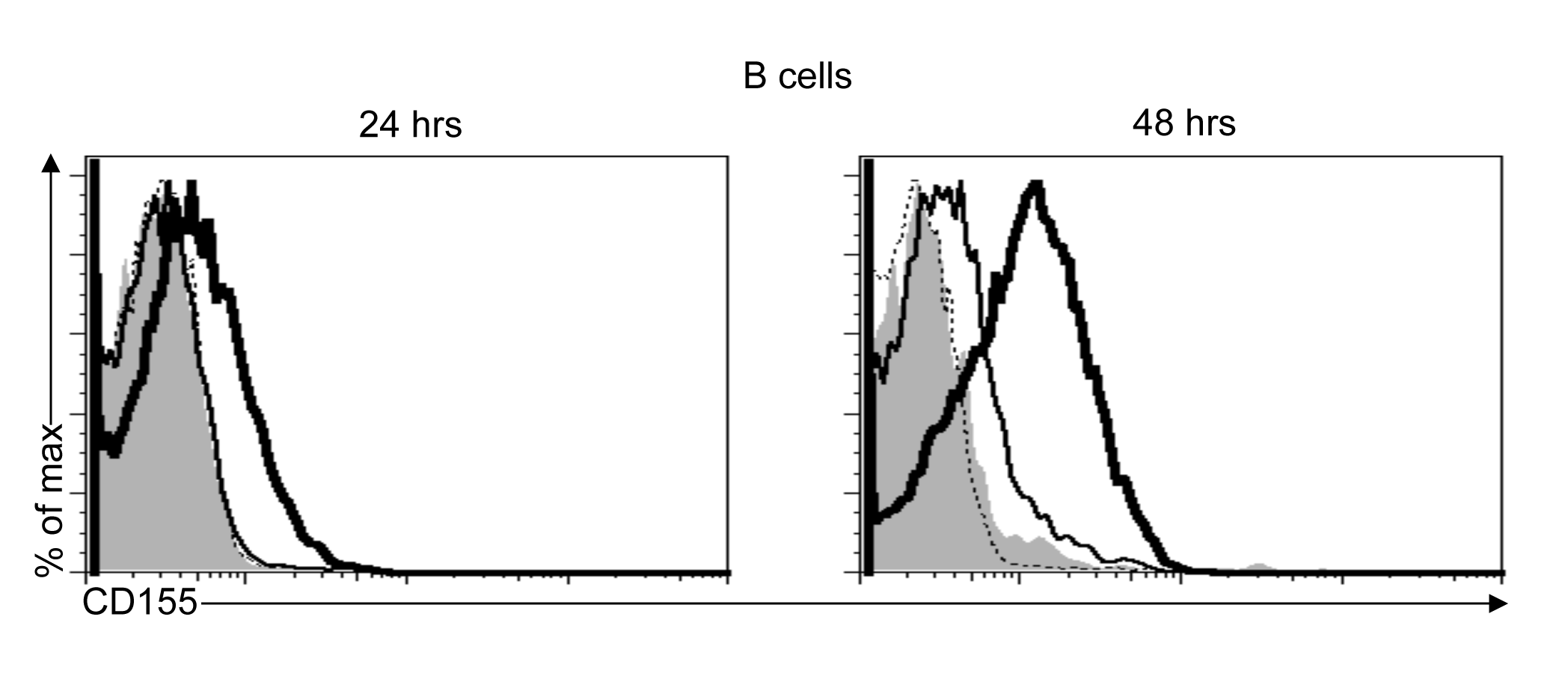

Supplement: Figure S1 — B cells show a delayed kinetics of CD155 upregulation in response to TLR agonists. B cells purified from the spleens of WT mice were treated with control (thin line) or 1 µM CpG (thick line) for the indicated times. Subsequently, cells were analyzed for CD155 expression by flow cytometry. Filled histograms and dotted lines represent control and CpG-treated cells stained with isotype control antibodies. Data are representative of three independent experiments. (TIFF) [file pone.0054406.s001.tif]

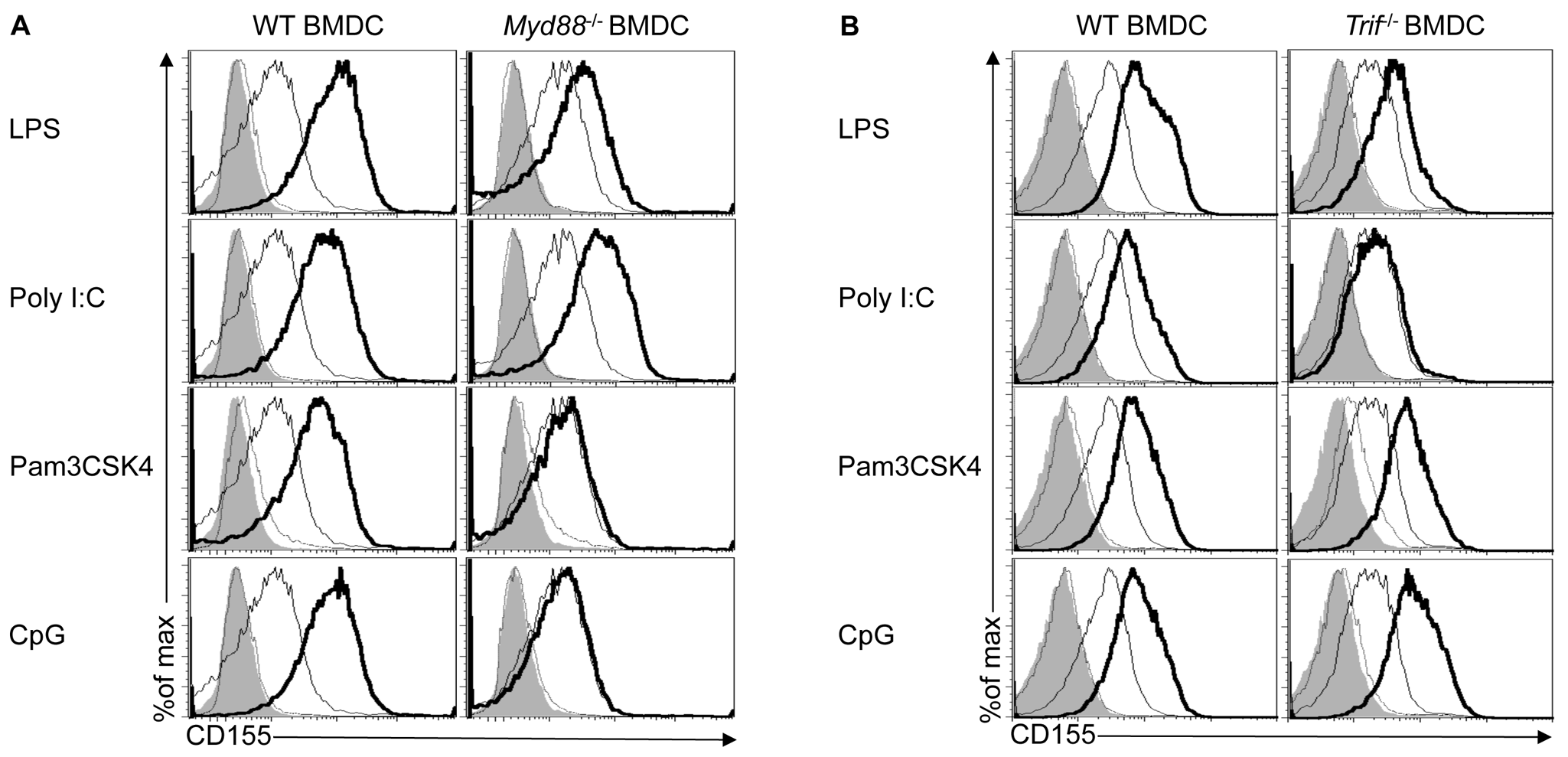

Supplement: Figure S2 — CD155 upregulation in response to TLR agonists depends on MYD88 and TRIF. Replicate staining of BMDCs derived from Myd88 −/− (A) and Trif −/− (B) mice is shown. (TIFF) [file pone.0054406.s002.tiff]

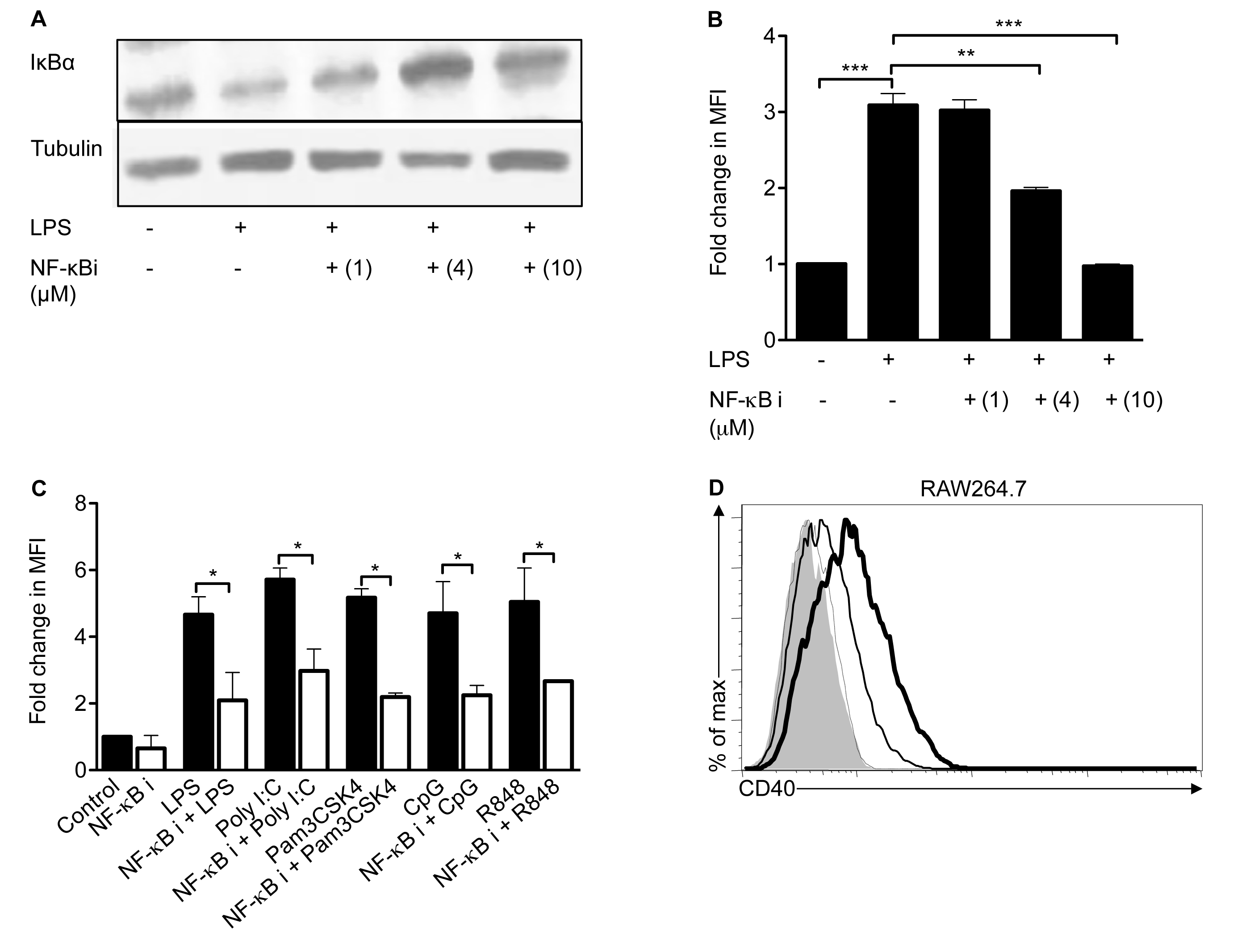

Supplement: Figure S3 — NF-κB inhibitor BMS-345541 blocks TLR-induced CD155 upregulation. (A) RAW 264.7 cells were treated with BMS-345541 for 1 hr followed by 1 µg/ml LPS for 5 hrs. NF-κB activation was detected by western blotting as a decrease in IκBα levels. Tubulin levels were used as a loading control. (B) Analysis of CD155 expression on RAW264.7 cells shown in figure 3A. (C) Analysis of CD155 expression on BMDMs shown in figure 3B. Groups shown in (B-C) were combined from three independent experiments and represent fold change of MFI ± SEM, * p<0.05, ** p<0.01, *** p<0.005. (D) RAW264.7 cells were treated with PBS (thin black line) or 20 ng/ml TNFα (thick black line) for 24 hrs and stained with CD40-specific antibody. Filled histograms and dotted line represent control and TNFα-treated cells stained with isotype control. Data are representative of two independent experiments. (TIFF) [file pone.0054406.s003.tiff]

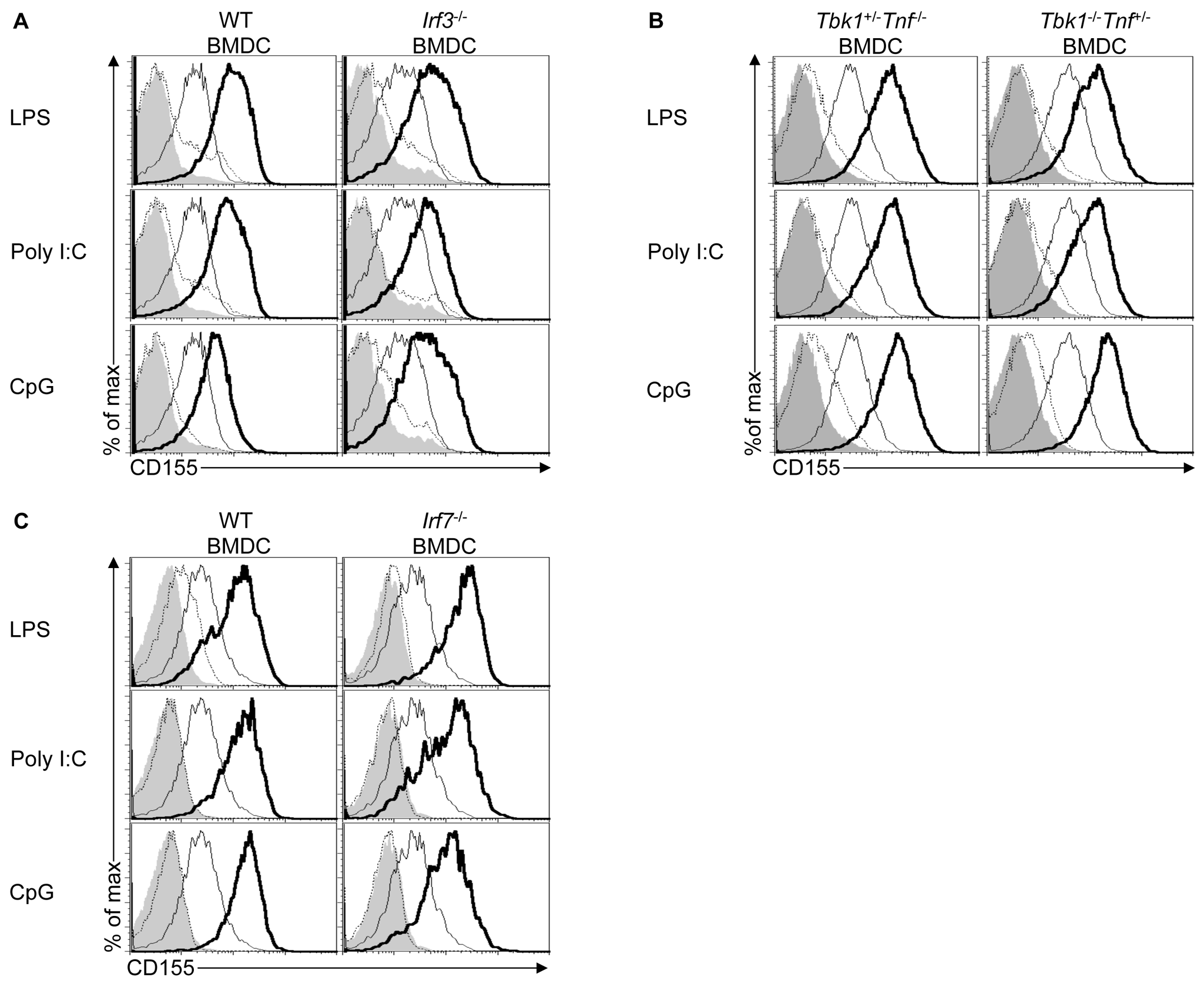

Supplement: Figure S4 — Induction of CD155 expression in response to TLR agonists depends on IRF3. Replicate staining of BMDCs derived from WT, Irf3 −/− (A), Tbk1 +/−;Tnf −/−, Tbk1 −/−;Tnf −/− (B) and Irf7−/− (C) mice. (TIFF) [file pone.0054406.s004.tiff]

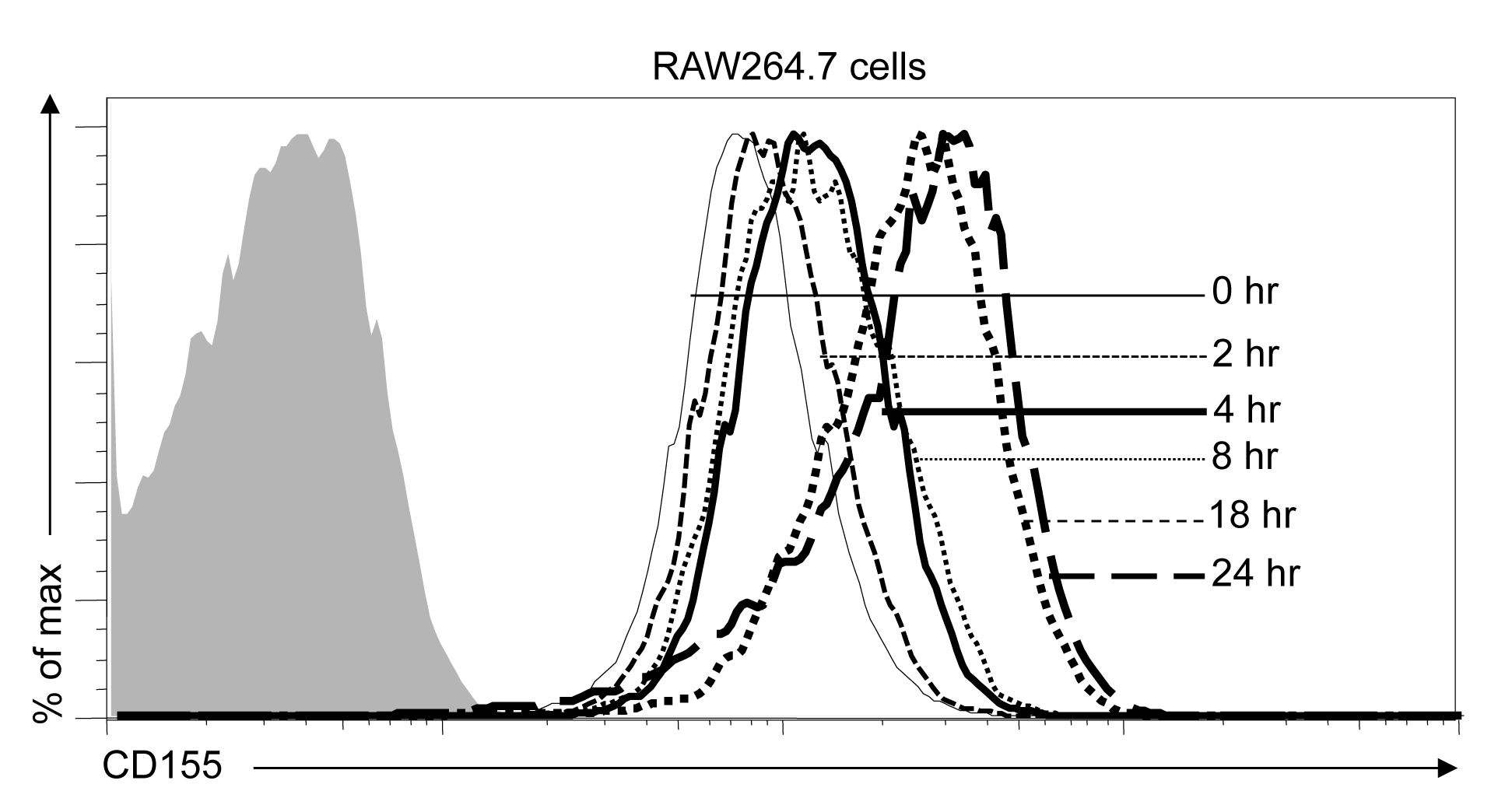

Supplement: Figure S5 — CD155 protein expression increases in response to TLR stimulation. Untreated RAW264.7 cells (thin line) or RAW264.7 cells were treated with 1 µg/ml LPS for 2 (thin dashed line), 4 (thin dotted line), 8 (thick line), 18 (thick dotted line) and 24 hrs (thick dashed line) and analyzed for CD155 expression by flow cytometry. Untreated cells stained with isotype control antibody are shown as filled histogram. Data are representative of three independent experiments. (TIFF) [file pone.0054406.s005.tiff]

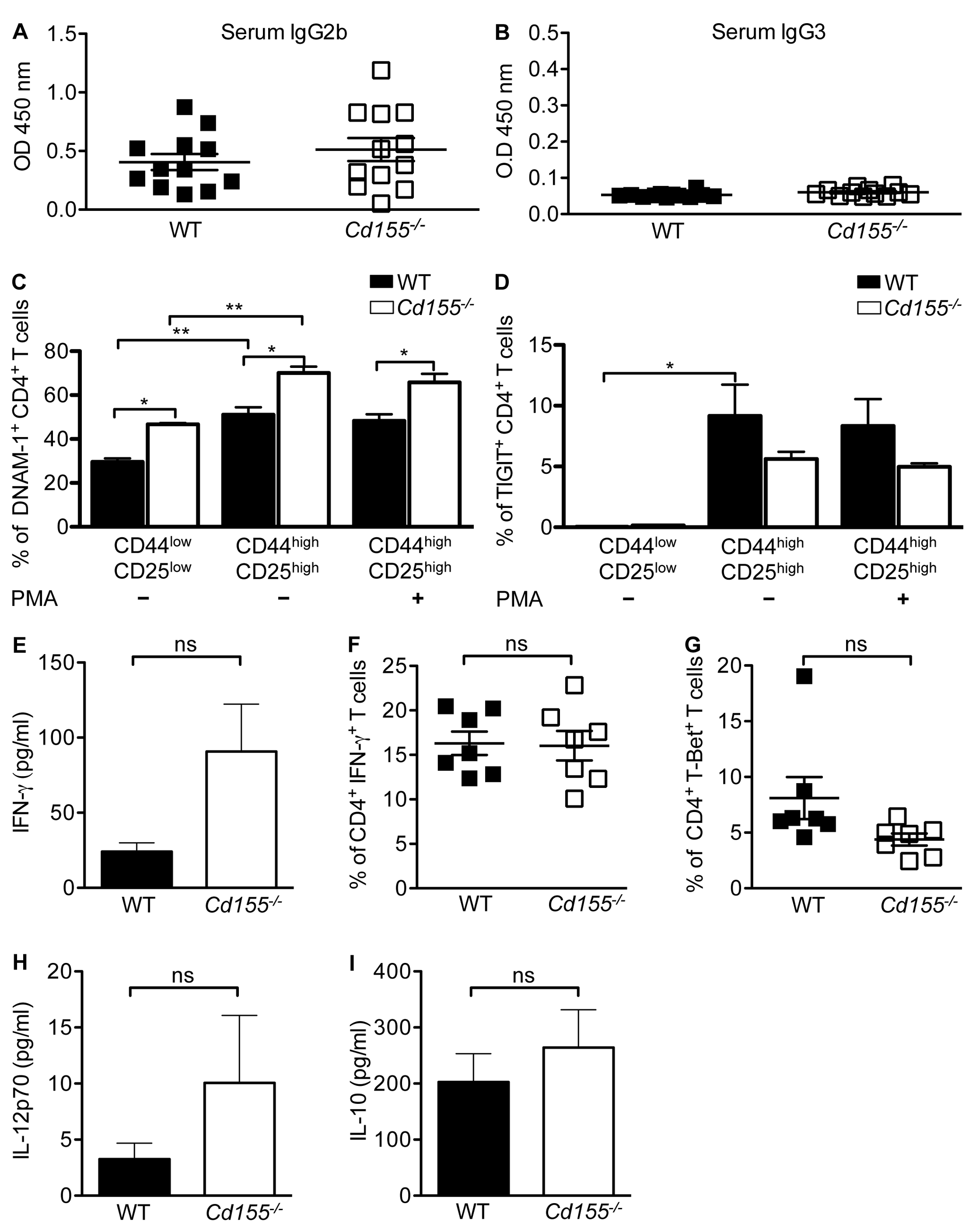

Supplement: Figure S6 — (A-B) Serum levels of OVA-specific IgG2b (A) and IgG3 (B) antibodies in Cd155−/− (n = 12) mice and WT (n = 12) littermates injected i.p. with OVA and CpG. Titers of OVA-specific antibodies were measured and analyzed as outlined in figure 7 . Combined data of three independent experiments are shown. (C-D) Analysis of DNAM-1 (C) and TIGIT (D) expression on CD3+CD4+CD25−CD44low and CD3+CD4+ CD44high CD4+ T cells from spleens of WT and Cd155−/− mice. Groups were compared using one-way ANOVA followed by Bonferroni post test and are expressed as means ± SEM, * p<0.05, ** p<0.01. (E) 5×10^6^ splenocytes of WT and Cd155−/− mice injected with OVA and CpG were cultured for 48 hrs, after which the culture supernatant was analyzed for IFN-γ levels by ELISA. Data are representative of two independent experiments. (F and G) Splenocytes of OVA and CpG-immunized WT and Cd155−/− mice (n = 7) were analyzed for intracellular IFN-γ (F) and T-Bet (G) expression by flow cytometry. (H and I) Cell culture supernatants from cultured spleen cells of OVA and CpG-immunized WT and Cd155−/− mice were analyzed for amounts of IL-12p70 (H) and IL-10 (I). Results shown in (E-I) were compared using Student’s t-test and are expressed as means ± SEM. ns indicates not significant. (TIFF) [file pone.0054406.s006.tiff]

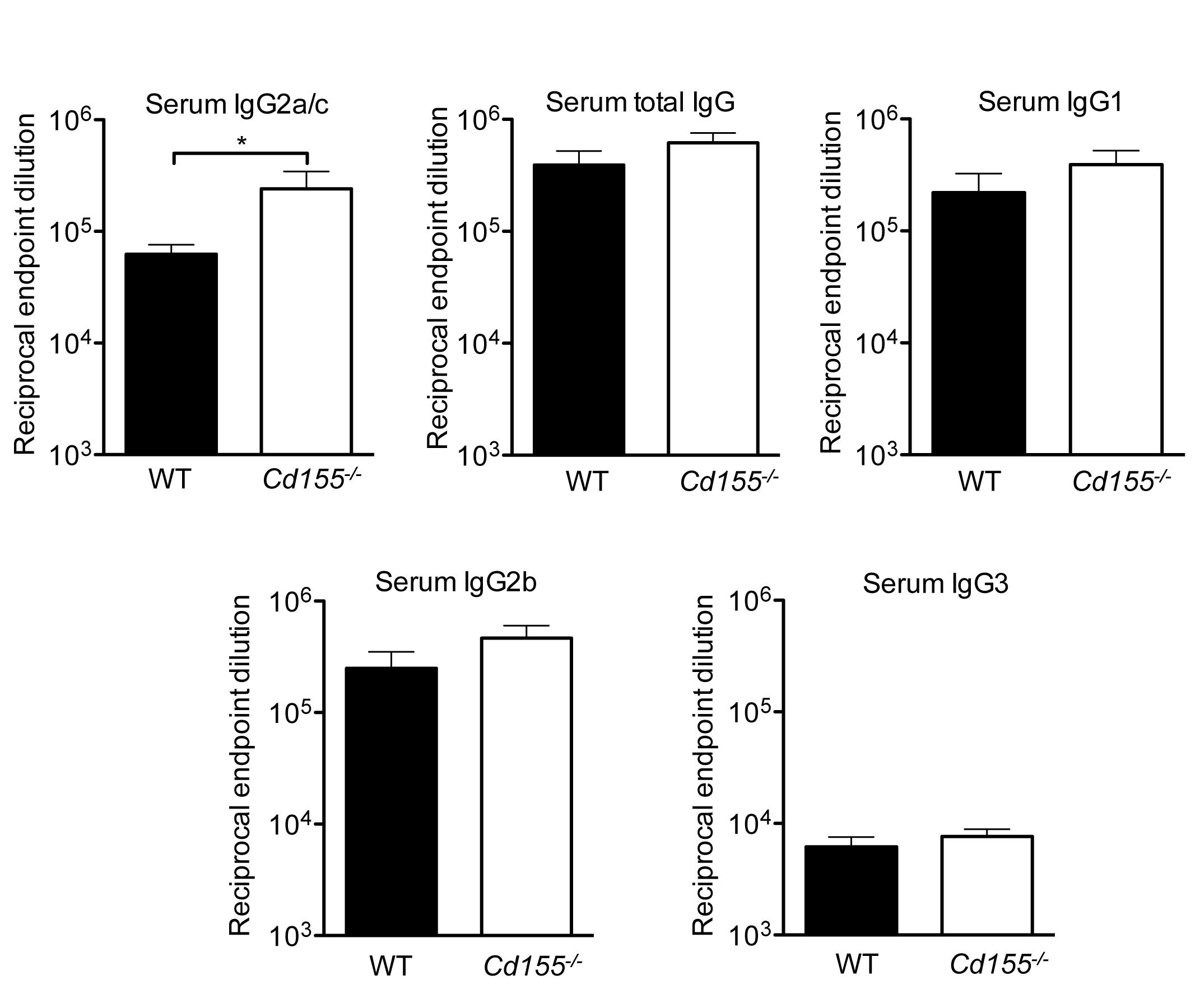

Supplement: Figure S7 — Increased IgG2a/c titers in OVA and CpG-immunized Cd155−/− mice when compared to WT mice. Results obtained in figure 7 (A) – (C) and Figure S6 (A) – (B) are shown as endpoint dilutions. Titers were calculated as the reciprocal of the last serum dilution that resulted in an OD at 490 nm above that of double the corresponding value obtained with the pre-immune serum. Data were compared using the Mann Whitney U test. * p<0.05. (TIFF) [file pone.0054406.s007.tiff]
